# The International Research Society of Spinal Deformities (IRSSD) and its contribution to science

**DOI:** 10.1186/1748-7161-4-28

**Published:** 2009-12-22

**Authors:** Keith M Bagnall, Theodoros B Grivas, Nathalie Alos, Marc Asher, Carl-Eric Aubin, Geoffrey R Burwell, Peter H Dangerfield, Thomas Edouard, Doug Hill, Edmond Lou, Alain Moreau, Joe O'Brien, Ian Stokes, Hans-Rudolf Weiss, Jim Raso

**Affiliations:** 1 Department of Anatomy, United Arab Emirates University, Faculty of Medicine and Health Sciences, Box 17666, Al Ain, Abu Dhabi, United Arab Emirates; 2 Trauma and Orthopaedic Department, "Tzanio" General Hospital of Piraeus, Greece; 3 Sainte-Justine University Hospital Research Center, Montréal, QC, Canada; 4 Kansas University Medical Center, 3901 Rainbow Blvd: MS 3017, Kansas City, KS 66160 USA; 5 Department of Mechanical Engineering, Ecole Polytechnique de Montréal, Station “Centre-Ville”, P.O. Box 6079, Montreal, QC, H3C 3A7, Canada; 6 'CAD Innovation in Orthopedic Engineering", NSERC-Medtronic Industrial, Spine Biomechanics, Ecole Polytechnique de Montréal, Station “Centre-Ville”, P.O. Box 6079, Montreal, QC, H3C 3A7, Canada; 7 Sainte-Justine University Hospital Center, P.O. Box 6079, Downtown Station, Montreal, Quebec H3C 3A7 Canada; 8 Centre for Spinal Studies and Surgery, Nottingham University Hospitals Trust, Queen's Medical Centre Campus, Nottingham, UK; 9 School of Biomedical Sciences, University of Liverpool, UK; 10 Viscogliosi Laboratory in Molecular Genetics of Musculoskeletal Diseases, Sainte-Justine University Hospital Research Center, Montréal, Qc, Canada; 11 Rehabilitation Technology, Glenrose Rehabilitation Hospital, 10230 111 Ave NW, Edmonton, Alberta, Canada; 12 Rehabilitation Technology, Glenrose Rehabilitation Hospital, 10230 111 Ave NW, Edmonton, Alberta, Canada; 13 Department of Stomatology, Faculty of Dentistry, Université de Montréal, Canada; 14 Department of Biochemistry, Faculty of Medicine, Université de Montréal, Canada; 15 National Scoliosis Foundation, 5 Cabot Place, Stoughton, MA 02072, USA; 16 University of Vermont,, Department of Orthopaedics and Rehabiliation, Burlington, VT 05405-0084, USA; 17 Orthopädie, Physikalische und Rehabilitative Medizin, Chirotherapie, Physikalische Therapie Alzeyer Str. 23, D-55457 Gensingen, Germany; 18 Glenrose Rehabilitation Hospital, 10230 111 Ave NW, Edmonton, Alberta, Canada

## Abstract

From the time of its initial, informal meetings starting in 1980 to its formal creation in 1990, the IRSSD has met on a bi-annual basis to discuss all aspects of the spine and associated deformities. It has encouraged open discussion on all topics and, in particular, has tried to be the seed-bed for new ideas. The members are spread around the world and include people from all areas of academia as well as the most important people, the patients themselves. Most notably, application of the ideas and results of the research has always been at the forefront of the discussions. This paper was conceived with the idea of evaluating the impact made by the IRSSD over the last 30 years in the various areas and is intended to create discussion for the upcoming meeting in Montreal regarding future focus: "*We are lost over the Atlantic Ocean but we are making good time."*

## Introduction

Ever since the days when deformities of the spine were considered from a topographical point of view at selective meetings of interested people, through the formal creation of the IRSSD itself and at its subsequent bi-annual meetings, there has always been a willingness for the expression of ideas and results that has encouraged the development of understanding of spinal deformities. An environment of friendliness and camaderie has certainly been created and maintained and there has always been an air tolerance, patience and understanding when it comes to scientific thought. Participants have always been encouraged to express their thoughts freely and discuss energetically while feeling comfortable doing so. However, while success might be measured by the size of the pile of small pebbles each contributed by the individual members, the question always remains as to whether or not the pile as a whole represents a coordinated whole and a significant contribution or is just simply a 'pile of small pebbles'. Consequently, this article has been compiled through a desire to evaluate the contribution made by the IRSSD and its members to the scientific literature - an exercise of 'introspection', so to speak.

From the programmes of the IRSSD as well as previous meetings, common themes and areas were identified and representative authors (we hope nobody is offended by not being invited) were asked to:

- focus on the changes that have occurred in a particular area and provide an assessment of what influence (if any) the Society has had on the health of society in general. We wanted people to read this article not simply put it on their shelves.

- make only brief summaries of the specific areas with brevity being a necessity (< 1000 words). The purpose of the paper was not to go through a detailed history of the area but to highlight the important progress that has been made - or not, as the case may be.

- include minimal references as the readers could find these elsewhere.

Contributions from representatives from all over the world have been included (in no particular order) as this reflects the spread and interests of the society. The different approaches and styles of the contributions to the requests are diverse and are of interest in themselves as they reflect differences in origin, personalities and points of view.

The contributors are to be applauded for their efforts because the request was not easy. There are many vehicles available for the dissemination of knowledge and the IRSSD is but one. Furthermore, the tapestry of research is complex and the interweaving of ideas makes it difficult to separate one issue from another. To isolate the contribution made specifically by the IRSSD is difficult but there is a line of thread in many areas that appears to have emerged over the years. To the reader, please enjoy!

## History of the IRSSD

The roots of the IRSSD go back to Vermont, 1980 and the application of moiré topography techniques to the study of children with scoliosis. Moiré topography is an optical technique that can be used to measure features of a surface. By 1979 a number of researchers in various parts of the world were developing tools based on this approach to assess the trunk deformity associated with scoliosis. The aim of the work was to develop objective measures that might be used to screen children for scoliosis as well as assess changes to the trunk as a result of surgery. Many of those in the field were present at the workshop organized by Drs Morey Moreland, Malcolm Pope and Gordon Armstrong. The inaugural workshop was divided in to 4 sessions: instrumentation, School Screening, Quantification and (as odd as it might seem today) computerization. Representatives attended from Japan, throughout the United States and Canada as well as from many European countries. It began a tradition that the IRSSD has continued of significant multidisciplinary participation. It also had a number of private sector participants. In the keynote address, Dr. Gordon Armstrong expressed the hope that from the meeting would come proposals to form an international society for moiré topography.

While a formal society was not immediately established, the group continued to meet biannually. The meeting in 1982 took place in Munster Germany hosted by Drs Drerup, Frobin and Hieholzer. Although there was still much to do with respect to techniques, the focus began to shift towards a search for the link between the external deformities of the trunk and the internal spinal misalignment. The idea that surface analysis might reduce the need for radiographs that require ionizing radiation began to form. The 1984 meeting was held in Oxford and was organized by Allan Turner-Smith and JD Harris. The challenge set out for the research community was "to provide an acceptable non-invasive means of characterising the different forms and severity of spinal deformity, to be able to predict the course it will take, and to show the effects of treatment." The hope for surface analysis had grown significantly. Alternative approaches to moiré topography were being developed and were first presented at this meeting by groups from Germany and the United Kingdom. In 1986, the meeting moved to Canada and was organized by Stokes, Pekelsky and Moreland. It was also the year that Professor Hiroshi Takasaki, one of the pioneers of the field, passed away suddenly. The last meeting to focus primarily on surface typography was held in Portugal in 1990. There was recognition at that meeting that although the analysis of surface features of the trunk resulting from scoliosis held valuable information, it had not developed into a standard clinical tool. Those present at this meeting committed to strengthening the group by constituting a new society, The International Research Society of Spinal Deformities (IRSSD). A meeting held in Montreal in 1992 combined the Spinal Deformity and Surface topography groups with biomechanics in an international symposium on 3-D Scoliotic Deformities. This meeting marked the beginning of the IRSSD group and a departure from the traditional focus on surface analysis to a consideration of the 3 dimensional nature of scoliosis and its biomechanics. Dr Morey Moreland was elected as the first president of the Society. The 3 dimensional nature of scoliosis continued as a theme for the 1994 meeting in Pescara Italy but began to change with the symposium held in Sweden in 1996 where the program included papers on the etiology and pathomechanisms of scoliosis.

The meeting in 1998 in Vermont broadened the mandate of the Society significantly. Under Ian Stokes direction, the program opened with talks on molecular biology and regulation of spinal growth - topics very far from where the group started 18 years before. In the years since, the Society has continued to cover a broad range of issues related to scoliosis. Subsequent meetings were held in Clermont Ferrand, France (2000), in Athens, Greece (2002), Vancouver, Canada (2004), Ghent, Belgium 2006 and Liverpool, England in 2008. Although the analysis of trunk asymmetries is no longer the main topic for discussion, it is still present in the program in the form of clinical applications and findings. The Society still is very much a multidisciplinary effort with a strong emphasis on the search for the underlying mechanisms that cause scoliosis as well as those that give rise to its disfiguring aspects and the clinical effectiveness of a wide range of interventions.

## Motivating Clinical Problems

The two most common spine deformities, scoliosis and kyphosis (more properly hyperkyphosis) have been recognized since antiquity. However, the first successful treatment for moderate to severe scoliosis and kyphosis, spinal arthrodesis, is a recent development. First successfully performed in 1911, it proved to be a difficult therapeutic methodology requiring decades to refine into a reliable operation. This served to focus treatment on the more severe cases and into the hands of orthopedic surgeons, limiting multi-disciplinary study.

The most common etiologies of kyphosis, fractures and tuberculosis revealed themselves relatively easily, and guided prevention and treatment. The same has not been true for scoliosis. For decades poliomyelitis residuals were blamed. However, well before the advent of the successful inactivated vaccine in 1955 removed all doubt, it was realized that most scoliosis was without known cause. The cause (or causes) of this condition, usually of mild to moderate severity, has stubbornly eluded detection, as has the development of universally accepted and obviously effective non-operative treatment.

There can be no doubt that this adolescent, also known as late-onset, idiopathic scoliosis was and is the clinical problem that motivated the formation and motivates the continuation of the IRSSD. It is an unequalled forum for information exchange for a wide spectrum of scientists and clinicians all interested in the puzzle that is idiopathic scoliosis.

Drawings and photographs done before the invention of radiographs in 1895 clearly depicted scoliosis as a three dimensional trunk deformity. Drawings did the same for the spine deformity. Radiographs however reduced visualization of the spine to two planes and largely ignored the trunk deformity. The need to quantify scoliosis as a three-dimensional deformity and advances in computer technology led in the 1980's to the parallel development of trunk surface topography and three-dimensional radiographic spine studies. Initially separate movements; it was desirable and probably inevitable that they come together.

It cannot be said that studies utilizing these techniques have produced a breakthrough in the understanding of idiopathic scoliosis. Even today the techniques have not been found beneficial in practice and are rarely used outside of research setting. However, as the result of these studies and the resulting discussion and collaborations, scoliosis is now widely recognized as a three-dimensional deformity. This has fostered better clinical trunk measurements and better analysis of biplanar radiographs.

Surface topography, anthropometric, stability and electro diagnostic studies have shown these persons to have some subtle but measurable asymmetries; more than comparable normal persons. The same seems to be true of patients with congenital scoliosis, suggesting the asymmetries are more related to the deformity than to the etiology.

Biplanar radiographic images are still the standard for determining the scoliosis deformity phenotype and its severity. Global kyphosis has been identified as a risk factor for associated neural axis abnormalities. Left apex thoracic curves are especially associated with this risk. Addressing the neural axis abnormality, e.g. Chiari 1 malformation and syringomyelia sometimes results in curve reduction and for those patients undergoing instrumentation and arthrodesis, a safer operation. Whether or not a three-dimensional deformity classification system that adds any deeper insights into the pathogenesis of idiopathic scoliosis can be developed remains to be seen.

While the search for etiology or etiologies remains as elusive as ever, these studies have substantially altered thinking about treatment methods to reduce the scoliosis deformity. Thought focused on vertical translational loads, principally distraction has been replaced by thought utilizing combinations of translational and angular loads and counter-loads, addressing the deformity three-dimensionally. This is true for both bracing and surgery. Perhaps fostering this thinking is the greatest clinical contribution of three dimensional studies to date.

Idiopathic scoliosis is both the most common spine deformity and generally it's least severe. A missing part of the treatment puzzle is a natural history study providing guidance for the most beneficial treatment selection at the tipping point between surgery and non-surgery. It is at this point that the health-related quality of life questionnaires, reported on at some of the more recent meetings, may be helpful in selecting treatment. Once crossed, the surgery bridge cannot be re-crossed.

The meetings have always focused on adolescent/late-onset idiopathic scoliosis. In fact, at the first meeting (1992) bringing the surface topography and three-dimensional radiography groups together, there wasn't a single presentation on scoliosis of other etiology. Since then there have been a few papers about other spine problems at each meeting. These problems include hyperkyphosis, osteoporotic fractures, flat back, spine aging, low back pain, spondylolysis and spondylolisthesis, infantile scoliosis, Scheuermann's, adult scoliosis, myelomeningocele, Duchene muscular dystrophy, congenital scoliosis and Prader-Willi syndrome. Though usually related to treatment, a few presentations have focused on the underlying pathology. However, these presentation have served to keep the meetings from becoming too narrow, one of the problems that led to the formation of the IRSSD in the first place!

The biannual IRSSD meetings continue the very useful service of bringing together clinicians with research and clinical scientists of diverse scientific and backgrounds, practices and viewpoints. Although to date idiopathic scoliosis remains just that, it is doubtful that anyone "knowledgeable" about idiopathic scoliosis is surprised. Contemplating the scoliosis puzzle can make you a little crazy! Perhaps like studying fever before knowledge of microbes.

## Surgical methods

The involvement of spine surgeons from all over the word and especially of some members of SRS in IRSSD meetings was very important. It gave them the opportunity to be acquainted with the principles for which this Society was founded.

The development of IRSSD from a society focusing on surface topography and other issues of biomedical engineering contributed to the analysis of the outcomes of the surgical treatment of spinal deformities from a new point of view, (3-D concept and surface studies). The surface topography started to be used as a critical outcome to assess the efficiency of the surgery. For example it is worth mentioning the work of Professor Suzuki on the issue, the ISIS apparatus developed in UK and similar surface documentation devises used in several places. Previously the radiological assessment was the sole gold standard outcome of the results of the surgical treatment. The implications of the utility of such an approach are numerous, including the insight in scoliosis aetiology. Useful information was derived from the assessment of the various instrumentations used for the correction of the spinal deformity.

The involvement of biomedical engineers in IRSSD meetings (C-E Aubin, Wafa Skalli, to name some), was very fruitful as well. Mathematical models and finite element analysis of the various constructs or operations gave a better insight of the surgical approaches used. In this regard the Canadian and the French biomedical engineers school contributed enormously.

New surgical approaches, (concave periapical rib shortening) based on a long standing research program on scoliosis aetiology, as this of Professor John Sevastikoglou (John Sevastik) were presented and discussed in these meetings.

New instruments, as the one for the safe insertion of transpedicular screws (Mac-Thiong JM, H.Labelle), in use with the late generation of instrumentation for the surgical correction of scoliosis were also presented.

The study and assessment of HRQoL instruments was one of the topics presented in the IRSSD meetings, an issue closely connected with the outcomes of surgical treatment of spinal deformity. Mark Asher was pioneering on this issue analyzing the SRS 22 questioner in connection to the surgical treatment of scoliosis.

The "Research into Spinal Deformities" (RISD) in the "Studies in Health Technology and Informatics" (SHTI) book series created a publication tradition with an impact on research in connection with surgical treatment. The number of citations for the group of presented papers dedicated to the surgical treatment appear in Table S1, Additional file 1 and Table S2, Additional File 2.

These statistics demonstrate the visibility that was achieved by the publication of the extended abstracts of the meetings, in the Medline indexed IOS Press books. All this material has also been sent to the US National Library of Medicine for indexing in PubMed. The benefits are obvious and the dissemination of this research worldwide had an impact on the surgical treatment for scoliosis and other spinal deformities.

## General Biomechanics

The IRSSD has always been an open society focusing on the problem (spinal and trunk deformity) and not the discipline of specialty. So one of its many contributions has been to widen the scope of understanding of scoliosis from a biomechanical perspective. Biomechanics means the interaction of the living system (emphasise 'living') with mechanical phenomena (forces, motion, etc).

### Body shape, posture and movement

The origins of the IRSSD were meetings aimed at gaining understanding of body surface shape, and three-dimensional aspects of spinal and trunk deformities. The geometry and anatomy is fundamental to understanding deformity and its progression. The Society has been at the forefront of efforts to develop sensitive techniques to measure deformity (e.g. in screening and detection of asymmetry), and perhaps more importantly, the changes in a person's shape over time (detection of progression relative to significant thresholds when therapeutic interventions should be initiated). From the outset, the IRSSD has emphasized the three-dimensional nature of deformity, and has employed optical, ultrasound, low-dose stereo-radiography, and other innovative methods to define the shape of the spine, the ribcage and the body surface in three-dimensions. In the forth dimension (time), trunk movement is very important to a person's ability to function, as well as for the evaluation of a patient (e.g. in planning surgery). Also, gait studies, and postural control (balance) have been implicated in the understanding of the origin (aetiology) of scoliosis.

### Biomechanics of scoliosis aetiology

Understanding the aetiology of idiopathic scoliosis remains a major challenge, and even in deformities of known origin (e.g. neuromuscular and congenital scoliosis) the exact role of mechanical factors is evidently complex. However, it is clear that deformities develop and progress during the period of skeletal growth. The effects of altered mechanical loading on growth plates and other tissues has been an important topic for research by IRSSD members, especially in non-human animal studies involving altered sustained and variable loadings. Equally, it is essential to know how spinal and trunk tissues are loaded in a range of everyday activities, and how these loads (especially the high forces developed in muscles) are altered by spinal deformity. Furthermore, tissues remodel even after skeletal maturity. The ways in which increased as well as decreased motion, along with the effects of altered stresses influence tissue remodeling of remain poorly understood. In scoliosis, both the vertebrae and discs become laterally wedged, probably by very different mechanisms, and the apical region is thought to become less flexible, adding difficulty to surgical 'correction'.

### Biomechanical analysis and simulation of treatment

The scientific literature is replete with studies of flexibility and strength of spinal constructs reinforced by the enormous range of surgical instrumentations that are available. Although required to assure the safety and efficacy of these medical devices, these kinds of analyses only scratch the surface of understanding the effects of surgery and other treatment. Members of the IRSSD have been centrally involved in developing a better understanding of the mechanical aspects of these treatment interventions. Collaborations between engineers, surgeons, and operating room personnel have produced intricate systems to measure intra-operative forces and to document the effects of surgical manoeuvers. In conjunction with these descriptive studies, analytical models have been developed to simulate surgery so that the outcomes of specific strategies on specific patients can be explored safely in advance. The variables include the patient positioning during surgery, the instrumentation and the exact manoeuvres performed during its installation, as well as the specific details of a patient. The same principles have also been applied to optimizing brace treatment (as described in greater detail under 'Mathematical modeling' - Aubin)

### What's next?

While the Society's emphasis has been on idiopathic scoliosis because it is common and a major scientific challenge, there are other spinal deformities that require attention. Notable are the progressive spinal deformity in elderly people having weakened (osteoporotic) bone, and spondylolisthesis that can be initiated in the young (often after neural arch injury) as well as later in life (associated with degenerative changes). However, major challenges remain in understanding idiopathic scoliosis. Given the evident importance of growth and its mechanical modulation, a major advance would be to develop predictive models capable of identifying patients at risk for significant progression of deformity before it occurs. What are the differences between two 12-year olds both with a 20 degree Cobb angle, one of whom develops a rapidly progressing curve and one who does not? Are the differences biomechanical? Another hugely important clinical problem will be to resolve the continuing controversies over the efficacy of non-surgical treatment, especially bracing and exercise regimens. These goals will require additional basic research into functional anatomy and biomechanics (how the different tissues are stressed and strained in different activities), as well as the response of those tissues over time, in both young and older individuals. The IRSSD will continue to be a forum where people with biomechanical expertise communicate, learn from and collaborate with others having clinical, biological and physiological expertise, and those with any other expertise who can bring insights into these enormously challenging problems.

## Aetiopathogenesis of adolescent idiopathic scoliosis (AIS)

The last 30 years has seen the pragmatic development and successful application of surgical techniques for the treatment of subjects with AIS. Less so have been the advances in knowledge of aetiopathogenetic mechanisms for AIS. Only two concepts provide the surgeon and doctor with a theoretical basis for treatment: relative anterior spinal overgrowth (RASO) and biomechanical spinal growth modulation. A third pathogenetic concept, thoracospinal, suggests a surgical treatment - concave periapical rib shortening, which has yet to be fully evaluated. Other concepts of AIS pathogenesis that suggest manipulatable causes lack, as yet, clinical evaluation. The challenge still remains to establish, if possible, bespoke treatments for AIS based on some knowledge of causation of the trunk deformity in the individual patient.

### What are the problems in attempts to solve AIS aetiopathogenesis?

Most experts agree that the causes of AIS are multifactorial with no generally accepted theory of pathogenesis, reflecting shortcomings in our understanding of the complex biological and biomechanical processes involved in AIS pathogenesis. According to some, innovative thinking 'outside the box' is needed. A second limiting factor is the tendency to focus on established biological and biomechanical fields, many 'keeping close to the bone'. In 2008-9 two attempts were made to present a balanced review of several theories of AIS pathogenesis (SOSORT and POSNA). Both were influenced by activities of the IRSSD. The current need is for a larger information base using systems biology approaches and hypothesis-generation, which has already been initiated from within the IRSSD.

### Scope of AIS aetiopathogenetic research at IRSSD

While in the period 1980-88 aetiopathogenesis was not addressed by IRSSD, since 1990 novel solutions have been presented. High technological research including genetics, molecular biology, imaging and mathematical modelling have tested particular concepts and generated new theories of pathogenesis.

#### 1990 Lisbon

The Nottingham theory for the pathogenesis of idiopathic scoliosis involving the CNS, rib-vertebral asymmetry and unique human trunk axial rotations was stated by Geoffrey Burwell *et al *and published further in *Acta Orthop Belg*.

#### 1992 Montreal

(1) Thoracospinal concept of early development of idiopathic scoliosis was presented by John Sevastik at this and subsequent IRSSD meetings and in journals. The thoracospinal concept is supported by recent studies on breast size, vascular and peripheral nerve findings. It is now integrated with the double neuro-osseous theory (2009).

(2) Ian Stokes presented evidence against both rib asymmetric growth and vertebral growth stress modulation as responsible for scoliosis progression. At subsequent IRSSD Meetings and in journals, including an IBSE electronic focus group (EFG) published in *Scoliosis*, Stokes, in extensive research, has supported the theory that asymmetric loading leads to vertebral wedging.

#### 1994 Pescara

Reporting on gait analysis of AIS patients, Peter Dangerfield supported the concept that pelvic and spinal movements are important in the causation of scoliosis (Nottingham concept).

#### 1996 Stockholm

(1) Peter Dangerfield presented longitudinal MR data on lumbar spinal length of healthy adult males (Pescara) and females (Stockholm) moving between recumbency and upright position. Changes in spinal length and sexual dimorphism were attributed to adapting disc water content.

(2) Ashley Cole presented anthropometric findings on preoperative AIS patients reporting a large extrathoracic skeleton (limbs) and upper arm length asymmetry. The general skeletal overgrowth in AIS girls is supported by subsequent findings in Hong Kong which gives pathogenetic significance (RASO) to this skeletal overgrowth.

(3) Caroline Goldberg presented her concept of developmental stability and AIS, now published in journals.

#### 2000 Clermont-Ferrand

(1) The neuro-osseous timing of maturation (*NOTOM*) concept to explain the female susceptibility to progressive AIS in relation to the somatic nervous system was declared by Geoffrey Burwell and later evaluated with Peter Dangerfield in relation to ballet dancers and rhythmic gymnasts (Athens IRSSD).

(2) Theo Grivas reported observations on menarche and AIS, subsequently published in *Scoliosis*.

#### 2002 Athens

(1) Georgios Kapetanos addressed the question: Is labyrinthine dysfunction a causative factor in idiopathic scoliosis? Recently, Shi Lin and colleagues developed shape analysis of the vestibular system in AIS subjects.

(2) Theo Grivas evaluated rib-vertebra angles and the lateral spinal profile in AIS pathogenesis.

#### 2004 Vancouver

(1) Alain Moreau and colleagues presented their initial findings of melatonin-signaling dysfunction. These and subsequent findings, extended in journal papers, led to the conclusion that melatonin-signaling dysfunction detected in osteoblasts, myoblasts and lymphocytes is a decisive factor for the pathogenesis of AIS.

(2) Marianne McMaster presented statistical evidence that AIS and vertical spinous process asymmetry is positively related to the early introduction to swimming. This startling finding, presented again at the Ghent IRSSD, can be accommodated by the double neuro-osseous theory.

#### 2006 Ghent

(1) A neurodevelopmental concept declared by Geoffrey Burwell *et al *led to collaborative preliminary research between Nottingham and Hong Kong workers involving MR brain scans of left thoracic AIS subjects, revealed reduced white matter density in the left internal capsule and corpus callosum (published in *Am J Neuroradiol)*.

(2) Winnie Chu and colleagues' paper on spinal cord tethering in AIS was published in *Spine *and an IBSE EFG in *Scoliosis*.

(3) Geoffrey Burwell's IRSSD presentations led to lectures at the 2008 SOSORT Conference (Theo Grivas), and the 2009 MILAN Conference (Stefano Negrini); and, in collaboration with Jack Cheng and colleagues in Hong Kong, to an article entitled "Top Theories for the Etiopathogenesis of Adolescent Idiopathic Scoliosis" to be published in *J Pediatr Orthop*.

#### 2008 Liverpool

The double neuro-osseous theory was formulated after revealing the different effects of body mass index (BMI) subsets on skeletal maturation, asymmetries and overgrowth in AIS and normal girls. Theo Grivas applied this BMI method to Greek children measured for trunk asymmetry and found an excess of severe trunk asymmetry associated with relatively lower BMI, published in *Scoliosis*. The double-neuro-osseous theory, recently published in *Scoliosis*, was used to interpret the findings.

## Conclusion

The IRSSD provides an ideal forum where ideas about aetiopathogenesis can be articulated, debated and recorded in the Proceedings Book of the Conference, with the important advantage that the abstracts are freely available on Medline. This is a valuable part of the activities of the IRSSD as it allows researchers and others early accesss to new ideas and concepts as they develop, something that does not happen for most scientific spinal meetings. Subsequent publication in journals is needed to influence progress. By then, IRSSD has provided one of its major roles - to facilitate the development and testing of novel concepts for the aetiopathogenesis of idiopathic scoliosis.

## Evolution of measurement techniques

Members of the IRSSD have been leaders in establishing and standardizing measurement techniques used to describe spinal deformities. The IRSSD membership and focus has broadened over its existence to include surgeons, clinicians, many branches of basic sciences, and engineers. The precursor to the IRSSD was the Moire Fringe Topography and Spinal Deformity biennial international symposium started in 1980. Two important decisions made then were to publish the papers from the meetings in a book and to meet regularly. The first meetings were relatively technical in nature. They brought together orthopaedic surgeons, scientists, engineers and clinicians from Japan, Germany, Austria, England, Sweden, Canada, and the US. This international scope of the IRSSD meeting has been maintained. The goal at the first meeting was to improve the methodology and to attain a better clinical understanding of surface shape changes in scoliosis. Radiation exposure was recognized as undesirable and the hope was that surface topography could reduce the number of radiographs required to monitor spinal deformities. Patient positioning was also recognized as important to make reliable measurements from the patient.

During subsequent meetings, attempts were made to standardize description of the asymmetries associated with spinal deformities. Studies were undertaken to relate surface features to radiographic findings, especially the Cobb angle and the rib hump angle. The primary concern for many adolescents with spinal deformity is the noticeable asymmetries of the trunk. Surface topography offered the ability to quantify these asymmetries, whereas radiographs were limited in this capacity. Surface topography equipment was expensive and required considerable technical skill to set-up and to operate. Measurements were labour intensive, descriptors were often qualitative, and the results were generally not available while the child was still at the clinic. This limited the wide-spread implementation and use of surface topography in the assessment of spinal deformities. With increasing computer power, commercially available surface topography systems such as ISIS, Quantec, and Formetric were developed to standardize patient positioning and descriptors of the deformity, and to reduce the labour and technical requirements. Individual parameters such as trunk rotation, shoulder and scapula angles, hump sum and Q-angle as well as composite scores of POTSI, cosmetic score and DAPI were developed to assess surface shape.

Over time, the IRSSD has added additional imaging modalities as well as genetics and aetiology, biomechanics, movement and posture, growth and metabolism, and assessment of treatment outcomes to its mandate. The IRSSD has advanced the development and use of measurement techniques in many of these topics. Terms from anatomy, biology and chemistry were added to the vocabulary at meetings. The IRSSD was a strong force behind research in 3D descriptors of spinal deformity leading to the Scoliosis Research Society terminology committee being established in 1999 to describe and standardize spinal deformity biomechanical terms. Terms were agreed upon to describe loads and displacements in local, spinal and global axis systems. The Spinal Deformity Study Group has identified a set of nineteen parameters describing salient radiographic features in a spinal deformity population. The parameters include measurements of curve size and location, spinal imbalance, sagittal plane alignment, vertebral rotation, T1 tilt, spondylolysis/spondylolysthesis, and skeletal age.

The digital age has provided much of the necessary infrastructure to allow the testing of hypotheses posed by IRSSD members. With the implementation of digital imaging modalities, radiation exposure required to monitor spinal deformities is considerably less than it was 30 years ago. However, the general public is much more sensitive and critical of any non-essential radiation exposure in growing children than ever before. There are now many additional tools beyond surface topography and radiographs to assess spinal deformity. Imaging modalities in use today, such as low dose 3D radiography, surgical navigation systems, ultrasound, and MRI can be used to model and visualize the spine in three dimensions.

Advances in image resolution and quality, monitor technology for display, increased storage capacity, increased bandwidth for transmission, and software tools for semi-automated measurement have dramatically changed how spinal deformity is assessed in scoliosis clinics. View boxes and 3 foot films have been replaced by monitors and software tools. The spine and surface shape can be viewed in three dimensions. In spite of this, there is not yet a suite of commonly accepted and used 3D parameters for the routine clinical assessment of children with scoliosis. The Cobb angle is still the mainstay tool used in spine clinics in deciding whether a child's condition has changed and what treatment should be offered. The appeal of the Cobb angle is its simplicity and familiarity rather than the belief of its superior ability adequately to describe the deformity.

IRSSD members have developed computer-based models based upon placement of applied forces and measurement tools to assess and predict brace outcomes. Monitoring of brace use to determine compliance and loading patterns has furthered our understanding of brace efficacy. Computer models of the spine based on position, forces and material properties coupled with the monitoring of forces and displacements during surgical manoeuvres have improved surgeon training, patient safety, and understanding of surgical methods.

Beyond measuring physical features, the impact of spinal deformities on quality of life has also been explored. The most widely accepted disease-specific tool is the Scoliosis Research Society (SRS) questionnaire and its predecessors. The suite of SRS questions has been translated and validated in many languages and cultures. Short and long-term affects of spinal deformity on function, pain, self-image, mental health, and satisfaction can be monitored. The impact of varied conservative or surgical treatments can be assessed over time.

As the IRSSD membership has broadened in scope, it has added many measurement techniques to describe and evaluate the causes and treatment of spinal deformity. However, the majority of these measurement techniques have found limited use beyond research environments. The gold standard Cobb angle is still the dominant measurement technique used in scoliosis clinics. It likely will not go away any time soon but has been complimented by additional measurements of asymmetry, forces, displacements and quality of life.

## Bracing for Scoliosis

Brace treatment is the most commonly used non-surgical treatment for scoliosis. However, its effectiveness is still controversial and the underlying biomechanical action is not fully understood. Starting from the very first IRSSD meeting, researchers have put much effort into investigating these two areas and have reported that the conflicting results in effectiveness are due to inconsistent inclusion criteria between different studies, different definitions related to brace effectiveness and unrecorded compliance of the patients. It has also been realized that the biomechanical action of the brace must be studied as a three-dimensional problem.

Regarding problems related to the 'inclusion criteria', some studies have included both male and female patients in their data analysis whereas other studies have included only the most compliant patients. Studies have also varied in the different age groups included for comparison. Regarding the 'treatment outcomes', most studies have used the amount of curve progression (Cobb angle) to determine the effectiveness. However, some studies have used 5 degrees or less of curve progression to indicate success whereas others have used progression of 6 degrees or more to count as failure. Some studies have even gone to the extent of identifying patients who have eventually required surgery as their criterion for failure. Without consistent definitions of all the parameters involved, it is difficult to evaluate the effectiveness of bracing especially between the different types of brace available and the relative performance of the different centers involved. Recently, standardization of criteria for AIS brace studies has been regulated by the Scoliosis Research Society (SRS) Committee on Bracing and Nonoperative Management. The application of the criteria will greatly enhance research protocols exploring the effectiveness of bracing and it is anticipated that much progress will be made in the near future as a consequence. The IRSSD can be proud of having contributed to the creation of these important criteria through its members and their work.

Regarding the issue of 'compliance', in the past, patients usually were simply asked if they used their brace and how often they used it. Some researchers added to this and looked for signs of wear and tear on the brace to determine how much the brace had been used, if at all. Some studies reported that the magnitude of the strap tension was highly correlated with the in-brace correction or the treatment outcomes. Such variance of this important parameter has stimulated IRSSD members and others to investigate the compliance issues to a greater extent. With improvements in electronics technology, many devices have been developed in recent years to monitor brace-wear compliance and this has added a much-needed dimension. Some devices use temperature or humidity sensors for measuring purposes while others use force switches and pressure sensors. However, most of the researchers have only recorded how much time the brace has been worn and do not record (or are unable to record) whether the brace has been worn correctly, especially in terms of pressure being applied. This is unfortunate because the absence of measurements of forces being applied may provide a much distorted view of overall compliance. Another important issue is the amount of time a brace is to be worn by the patient on a daily basis. Unfortunately, guidance given to the patient is generally based on 'clinical intuition'. The most commonly recommended time for wearing the brace is 23 hours per day. In recent years, the SRS has raised doubts as to whether part-time brace wearing is effective at all but even if part-time wearing is effective then the question arises: How many hours per day is sufficient? There is no doubt that prediction of the brace treatment outcomes is difficult, but the IRRSD members have reported that the brace treatment outcome depends on the risk of progression, the in-brace correction, and the compliance (both wear time and wear tightness). However, it is clear that there are still many questions in regards to bracing which require answers before major advancements can be made. For example: How do you define the optimal brace-wear tightness? The in-brace correction depends on curve flexibility which correlates highly with treatment outcomes, but how much correction is needed to provide the optimal results? What is the best way to determine the flexibility of the spine? Although bending radiographs can provide certain flexibility information, exposing growing children to additional radiation is undesirable and this would be a difficult study to design.

Understanding the biomechanical action of a brace is also of particular importance. Some IRSSD members (and others) believe that the Hueter-Volkmann principle contributes to the development of scoliosis with asymmetric loadings or compression force applied to the growth plates leading to wedging of the vertebral bodies. In theory, bracing a scoliotic curve should unload the growth plates on the concave side of the vertebral bodies near the apex of the curve but the evidence of the precise action of the brace based on the Hueter-Volkmann principle is still limited and an understanding of its action remains theoretical. Two other interesting and significant concepts to explain the actions of the brace have been discussed in the literature and at IRSSD meetings; one suggests that the brace provides mechanical support to the body (passive component) while the other suggests that the patient pulls her body away from pressure sites (active component) to correct the curve. Such diverse concepts illustrate the complexity of this problem but many of the IRSSD members believe that the most important focus of brace treatment is to provide the 3D correction and methodologies must be developed with this in mind.

Currently, there are still many questions with regards to brace treatment which remain to be answered. Orthotists certainly play a significant role in brace treatment regimens and their skill and experience clearly affect the design of the brace as well as the parameters related to its wear. It is well recognized that gender, skeletal age, curve type and the initial curve magnitudes affect the probability of progression and also the responsiveness to bracing. Similarly, the patient-compliance, in-brace correction and the curve flexibility also contribute to the outcome of the brace treatment. Support and encouragement from family members as well as peers are especially important during the brace treatment period and are recognized as vital to any success. Accordingly, to predict or evaluate the effectiveness of any brace treatment, knowledge of the compliance, in-brace correction, curve flexibility and risk of progression will play a significant role. Therefore, more research is required before the actual function and the effectiveness of bracing can truly be evaluated but, in the meantime, bracing certainly remains a viable and effective treatment strategy with much potential and remains a lively area for conversation among members of the IRSSD.

## Non-operative aspects and conservative treatment (including exercise and postural control techniques) of spinal disorders

The IRSSD developed from a society focusing on surface topography and other issues of biomedical engineering to an open forum for the discussion of all aspects of diagnosis and treatment of spinal deformities. From the very start it was open to biomedical engineers, anatomists, epidemiologists, geneticists, but also to spine surgeons, orthotists, physical rehabilitation specialists and physiotherapists. It was during the founding meeting in Pescara 1994, Italy, when Marc Asher, a well recognized surgeon and SRS member, during a discussion on conservative management suddenly asked for opinions on the efficiency of bracing. It was surprising to discover a surgeon who really wanted to learn more about conservative measures in the treatment of scoliosis. This was one of the most important historical moments for conservative treatment of spinal deformities and ignited a small flame which now burns brightly within the IRSSD. It also created the spark to develop SOSORT which would not have been established without this prior development under the umbrella of the IRSSD.

Physiotherapy treatment of spinal deformities is now well established and new indication guidelines have been widely accepted. It was the IRSSD which enabled the publication of new developments in this field particularly in its proceedings which have been listed in Pub Med (search for Stud Health Technol Inform, scoliosis). New developments in non-invasive treatments, especially of bracing, were first presented at an IRSSD meeting before subsequent results were published in other scientific journals.

Rehabilitation treatment strategies for a variety of spinal deformities as well as chronic pain have also been presented and discussed during IRSSD meetings as well as brace treatments of chronic low back pain and other rare conditions. Overall, the IRSSD has provided a very firm base from which conservative treatment strategies for spinal deformities could receive attention and where the initial distribution of any advancements could be made.

Today there is rapid development of conservative methods of treatment and a change of paradigm which has increased during the last two decades. When the IRSSD started in 1994 in Pescara, very few people in the scientific world believed in conservative treatment of patients with spinal deformities. Now, more than 15 years later, there is much evidence for the success of such conservative treatment and confidence in such strategies. So much so that surgery as a treatment option is being questioned more and more.

During the years, more and more specialists on conservative management, particularly Dr. Manuel Rigo from Barcelona and Dr. Stefano Negrini from Milano, as well as dedicated surgeons with broad interests in conservative management, such as Dr. Theo B. Grivas from Athens, Dr. Toru Maruyama from Tokyo and Dr. Tomasz Kotwicki from Poznan, have presented their work regularly at the biannual meetings of the IRSSD which have taken place in both Europe and North America. Afterwards the proceedings have been listed in Pub Med and, as such, the publications of this conservative group have gained a wide visibility (search for the authors mentioned above and Stud Health Technol Inform, scoliosis). More recently, other well recognized specialists in the field of conservative treatment of spinal deformities have presented their work during IRSSD meetings and their influence is growing.

When the off-shoot organisation of SOSORT had its 1st meeting in Barcelona with Dr. Manuel Rigo as the host, the main founding members were professionals who had met regularly at previous IRSSD conferences. Significant additions were Prof. Martha Hawes and Joe O'Brien, both of whom later played a major role within the SOSORT organisation.

In regards to conservative treatment strategies for spinal deformities, the IRSSD has been a multiprofessional, unbiased, open forum with open discussion. Scientists have been encouraged to contribute to common knowledge and been willing to learn more about a rare disease which still keeps secrets to be enlightened in the future. As a representative of this particular group of contributors I am glad and proud to have been part of it over all these years and would like to encourage its continued development in the future.

For some references for "Non-operative aspects and conservative treatment." please see Additional file 3.

## Metabolic and Hormonal Determinants of Adolescent Idiopathic Scoliosis

Adolescent idiopathic scoliosis (AIS) is the most common form of scoliosis and affects a significant number of young teenagers, mainly females (0.2-6% of the population). Despite extensive research for decades, the cause of AIS remains unknown. Several hypotheses have been postulated to explain the aetiology of AIS, including aspects of genetic, mechanical, neurological, muscular, biochemical and hormonal factors, resulting in the traditional paradigm that AIS is a multi-factorial disease with a genetic predisposition. In this contribution we have focused on the metabolic and hormonal determinants of AIS and the role of the IRSSD in this area.

Growth and sexual maturation are associated with the development and progression of scoliosis. It is well recognized that AIS primarily occurs in girls during the pubertal spurt and that girls with AIS have a growth pattern different from normal controls. These girls have lower body weight and body mass index (BMI), and higher corrected-height than healthy controls throughout the peri-pubertal growth period. Girls with AIS also present generalized lower bone mass and osteopenia in both the axial and peripheral skeletons.

### Melatonin signalling pathways

The neuroendocrine hypothesis involving a melatonin deficiency as the cause of AIS has generated great interest. This hypothesis stems from the fact that experimental pinealectomy in chicken, and in rats maintained in a bipedal mode, produces a scoliosis. More recently, similar results have been obtained in bipedal and quadrupedal C57BL/6J mice (a naturally, melatonin-deficient mouse strain). In these C57BL/6J mice, experimental scoliosis was induced without pinealectomy and melatonin treatment suppressed the development of scoliosis. Analysis of melatonin signal transduction in musculoskeletal tissues of AIS patients has demonstrated a defect occurring in a cell autonomous manner in different cell types isolated from AIS patients suffering from the most severe form of the disease. These results have led to a classification of AIS patients into three different functional groups depending on their response to melatonin, suggesting that the cause of AIS involves several genes. More specifically, molecular analysis has shown that melatonin signaling dysfunction is triggered by an increased phosphorylation of Gi proteins inactivating their function.

### Estrogens

In 2002, Inoue and colleagues reported that curve progression and severity of the scoliosis was associated with estrogen-receptor gene polymorphisms that are genetically determined. More recently, a cross-talk mechanism between 17-beta-estradiol and melatonin signaling in human AIS osteoblasts has been suggested as being involved. Accordingly, it is possible that the increased cAMP levels induced by melatonin can be corrected by the treatment of the cells with 17-beta-estradiol. In these circumstances the ability of estrogens to modulate G protein levels (Gs and Gi) might explain the incidence of scoliosis during puberty and the higher incidence in girls.

### Leptin

Leptin, the adipocyte-specific protein of the *ob *gene, has provided the first physiologic links to a regulatory system controlling body mass and bone mass. Serum leptin levels have been found to be positively correlated with BMI and bone mass. Moreover, leptin, whose level is much higher in girls than in boys, has been suggested to play a role in pubertal growth of girls, including onset of menarche, body growth, and development.

Marked decreases in circulating levels of leptin have been found in girls with AIS in comparison to healthy girls. Leptin levels are correlated strongly with body weight and BMI, and positively with other growth parameters, such as chronologic age, menstrual status, and Risser sign. Moreover, associations between leptin and bone mineral density have been demonstrated. These results suggest that decreased leptin levels might play an important role in the lower body and bone mass found in AIS girls.

It has also been suggested that truncal asymmetry is caused by a genetically-determined, selectively increased sensitivity (up-regulation, i.e. increased sensitivity) of the hypothalamus to leptin with asymmetry as an adverse response to stress, increased by lower circulating leptin levels associated with relatively lower BMI. This hypothalamic functional asymmetry is expressed via the sympathetic nervous system bilaterally to produce left-right asymmetry in ribs and/or vertebrae leading to severe truncal asymmetry.

### Calmodulin

Platelet calmodulin is a calcium-receptor protein closely related to calcium transport and muscle contractility. Lowe and colleagues have shown that patients with progressive curves secondary to adolescent idiopathic scoliosis demonstrated increasing platelet calmodulin levels that closely followed curve progression with growth increase. Calmodulin levels usually decreased in patients undergoing spine fusion or brace treatment. Altered paraspinal muscle activity may explain the relationship between platelet calmodulin level changes and Cobb angle changes in AIS with calmodulin acting as a systemic mediator of tissues having a contractile system. It has been hypothesized that serial platelet calmodulin levels may serve as a marker for curve progression in adolescent idiopathic scoliosis.

### Osteopontin

More recently, the study of the molecular changes occurring in pinealectomized chickens also revealed an aberrant production of osteopontin (OPN), a multifunctional cytokine, at the mRNA and protein levels, in paraspinal muscles of scoliotic chickens. This initial observation, presented at 2009 SRS meeting, San Antonio, has led to assessment of the role of OPN in AIS patients. The clinical relevance of this molecule is strengthened by the fact that plasma OPN levels in patients with AIS were higher when compared to healthy control subjects, and also correlate with curve severity. Indeed, the data suggest that plasma OPN levels could be used to discriminate between scoliotic patients with moderate spinal deformities (<45°) and those exhibiting severe deformities (>45°). Besides OPN being known as a transcriptional target of melatonin, studies in genetically modified mice have shown that scoliosis formation and curve progression proceed through OPN-CD44 signaling, since the inactivation of either the OPN or CD44 encoding gene prevented scoliosis development in bipedal C57Bl/6J mice.

This brief description of the line of thread through the hormonal research area related to scoliosis has focused on more recent data. There have been many vehicles used to carry and disseminate this information and the IRSSD has played a significant role in its delivery right from the beginning and throughout its development. While the characteristics of scoliosis has always suggested a theoretical, underlying 'hormonal' cause, the IRSSD with its relaxed atmosphere, open forums and encouragement to incorporate all aspects of study, has contributed in a large way to the development of this research area which has shown so much promise and produced such exciting discoveries particularly in recent years.

For some references for "Metabolic and Hormonal Determinants of Adolescent Idiopathic Scoliosis" please see Additional file 4.

## Finite Element Modeling of Scoliosis

Scoliosis is a complex 3D deformity of the spine whose biomechanics is difficult to comprehend using only radiographs or external measurements. However, it is obvious that its pathomechanism has an important biomechanical component. Biomechanical factors may arise from different sources such as dysfunction in the control of muscles eliciting balance problems and asymmetric loads on the skeleton, mechanobiological dysfunctions, growth alterations, etc. These biomechanical elements cause progressive deformation of the intervertebral disks, vertebrae, ribs, and pelvis. Conservative treatment such as bracing and minimally-invasive fusionless techniques aim at applying forces on the patient's skeleton in order to influence the deformation process and restore the spine alignment.

Finite element (FE) analysis for the study of scoliosis biomechanics has greatly evolved over the years due to the exponential progress of computer capabilities and modeling tools, and the increase of knowledge in complementary disciplines, in part due to collaboration between members of the IRSSD. Biomechanical/computer models are of practical interest because they provide the possibility of simulating an unlimited number of variables to investigate scoliosis biomechanics (pathomechanisms, spine growth, spine mechanisms and stability, coupling interaction between the spine, rib cage, pelvis, etc.), to predict the resulting shape of the spine in response to the application of a treatment as well as to optimize the treatment.

Early FE models of the osseo-ligamentous trunk system were very simple (e.g. Andriacchi and Schultz in the 1970's) and were used as a first attempt to analyze scoliosis and treatment biomechanics on generic scoliotic shapes. Over the years, the models have been refined to include patient-specific geometry using 3D reconstruction from bi-planar radiographs (I. Stokes, C.E. Aubin, W. Skalli) and patient-specific mechanical properties and boundary conditions using flexible tests and optimization processes (Y. Petit, V. Lafage, JP Little). The FE models were refined further to include the muscles (I. Stokes) and motor control (M. Beauséjour, V. Pomero), growth deformation processes which included the Hueter Volkman principles (I. Stokes, I. Villemure), and lower limbs (C Driscoll). The models also evolved into more detailed representations to include a better definition of the anatomical constituents like the bones and articulations (M. El-Rich, A. Sevrain), growth plates (P.L. Sylvestre), soft tissues (JP Little), bone property distribution (A Garo), etc.

FE models were refined and exploited to analyze many different scoliosis applications. For instance, FE models were used to test scoliosis pathomechanism hypotheses: asymmetric growth of the rib cage (I. Stokes), neuro-central joint asymmetrical growth (A.M. Huynh), abnormal anterior spine growth profiles (S. Lin), muscle impairment (A.M. Huynh), and concave-convex biases in the progression of scoliosis (M. Driscoll). FE modeling also was used to investigate the coupled mechanisms between the scoliotic spine and the rib cage subjected to loads corresponding to a brace (C.E. Aubin), as well as to study brace biomechanics (D. Périé, C.E. Aubin), and optimize brace effectiveness and optimal orthotic loads (G.T. Wynarski, D. Gignac, C.E. Aubin). More recently, a brace simulator using a parametric FE model has allowed virtual testing of many design options and optimizing their effectiveness (J. Clin). Biomechanical models also were used to simulate surgical instrumentation. Early models allowed reproduction of main individual (and simplified) maneuvers of the scoliosis surgery (I. Stokes, F. Poulin, V. Lafage) and simulation of costoplasties (J. Carrier, L. Grealou), while more recent models have allowed more detailed simulations of instrumentation strategies (Y. Lafon, R. Dumas, C.E. Aubin, X. Wang), simulation of patient positioning (K. Duke, C. Driscoll), and analysis of surgical instrumentation strategies (M. Robitaille), and optimization (Y. Majdouline). A spine surgery simulator integrating a computer model in a surgeon-friendly interface or a virtual environment now allows the surgeon to test different instrumentation strategies him/herself (C.E. Aubin, M. Côté).

FE models can yield valuable insights into the associated biomechanics of scoliosis and in the development of better treatment strategies even though particular attention should be paid when exploiting and interpreting simulation results, in order that they are not used beyond their scope of validity and application limitations. In the next few years, progress in understanding scoliosis from a biomechanical point of view is likely to be made by combining this sophisticated approach with other knowledge provided by complementary fields of research. It is anticipated that the IRSSD will continue to play a major role in facilitating this progress.

## The Value of the IRSSD ~A Patient's Perspective

In 1996 I bicycled across the U.S. from San Francisco to Boston to raise awareness about scoliosis and to solicit donations to fund etiology research. The funding was specifically for Dr. Nancy Miller's relatively new genetic study at Johns Hopkins University. Our "Cycle for the Cause" event enabled us to provide initial seed funding of $30,000 to Dr. Miller and also recruit 1,200 people for her project making this the largest genetic study at the time. I met Dr. Miller at the Scoliosis Research Society (SRS) meeting and learned of her work. I had a strong personal interest in this genetic research due to the fact that I am a patient who has had four surgeries for scoliosis and we have twelve family members within three generations affected by this spinal deformity, including three of my five children.

The primary motivation for the bike ride however was not my personal interest in Dr. Miller's work but rather a much broader recognition that the patient pathway for scoliosis was physically, emotionally, and economically burdensome for children and families and there seemed to be no end in sight. After fifteen years on the Board of the National Scoliosis Foundation I had become the second President leading this patient organization. In my capacity I spoke to more than 1,000 patients a year answering their questions and giving them the support and information they sought to make critical health care decisions for their children. We were celebrating our twentieth anniversary and upon reflection of this momentous occasion I realized how rewarding, yet utterly frustrating this task was.

Notwithstanding the time and attention given to this condition by Hippocrates, Galen, Andry, Cobb, Moe and many other brilliant minds for more than two millennia, our knowledge base was still woefully lacking. The reality was that in the vast majority of the time we did not know who was going to get scoliosis; we did not know why they had it; we didn't know how to prevent it; we often watched it turn into a deformity; we weren't sure if it would progress; we didn't know how far it would progress; we were unsure if we could truly stop the progression; we employed diagnostic and observation methods that were harmful; our non-operative methods to prevent progression had negative clinical implications and questionable results; and our accepted standard of corrective care required abnormal reconstruction of the spine, often with significant sequelae, that did little to improve the signs and symptoms of the condition, and had unknown long term outcomes and exponentially increasing costs.

In the meantime, the research in the peer reviewed literature seemed to be plagued with contradiction due to the acceptance of "idiopathic" as a legitimate diagnosis and the resultant inability to truly compare apples to apples. Moreover, the focus appeared to be centered increasingly on who had the better widget rather than gaining a more comprehensive understanding of this neuromusculoskeletal disorder. Our vision was to one day close our organization because the need was no longer there, yet we saw that the SRS was evolving away from the original mission of "finding the cause, prevention, and cure of scoliosis" and we feared that this would mean we would never close but rather deal with constant unknowns and increasing burdens and frustrations for families.

After the bike ride, and my fourth surgery, I attended my first IRSSD meeting held in Burlington, VT in 1998. I had met the local host, Ian Stokes, at several SRS meetings and he extended an offer to present a poster about our organization. I enjoyed Ian's presentations and was intrigued by his "Vicious Cycle" theory so I decided to take him up on his offer. I will never forget that meeting. On one hand, there was a comfort level of seeing well known orthopedic surgeons from the SRS like Dr. Marc Asher, yet on the other hand there were new faces like Hans Rudolph Weiss from Germany espousing a physical therapy method called Schroth; Dr. Charles Rivard from Montreal passionately telling me about the concept of dynamic bracing; surgeons and scientists from Europe exploring new non-invasive diagnostic techniques; engineers highlighting the biomechanics and three dimensionality of spinal deformity; and academics from England speaking of new theories of etiology and patho-mechanisms. The presentations were less formal and longer than I was used to at the SRS, but the depth of multidiscipline discussion and the level of open dialogue was exhilarating. I came away from that meeting with a renewed hope that one day my grandchildren may be spared the legacy of a curving twisting spine, and that finding the cause, prevention and cure for scoliosis was a shared vision and a dream that was still alive.

Since that first meeting in Vermont my appreciation for the value of the IRSSD and its members has only increased. And my hope for a better understanding of this condition and a more definitive, harmless, cost-effective, evidenced-based patient pathway with a paradigm change from correction to prevention continues to grow. I have great respect for the "think tanks" in Montreal, Alberta, Hong Kong, England and others throughout the world who remain steadfast in their pursuit of scoliosis knowledge, and who come together every two years at the IRSSD to collaborate in a global, multidisciplinary summit to advance that knowledge. It is through this effort that we will one day truly help scoliosis patients by finding the cure, and we can finally celebrate by joyously closing the National Scoliosis Foundation.

## Summary and Future

In one form or another, the IRSSD has been active for ~25 years. During that time it has encouraged open discussion in all areas of the spine and encouraged contribution, involvement and interaction among people from all over the world. The IRSSD has focused its attention on all areas of understanding and placed emphasis on encouraging and providing a suitable environment for inter-disciplinary discussion. The separate contributions contained in this article reflect the different approaches to the same instructions, the different styles of response, and the different progress made in almost all areas related to the spine. An overall summary of the reading might suggest that the IRSSD seems to have succeeded to a large extent and is to be encouraged to continue to develop as its contributions appear to have been significant and important. It might be most appropriate to conclude not with any definitive statements where we pat ourselves on the back but with a series of questions in the way that reflects the nature of the identity of the IRSSD:

- the IRSSD has remained a small and intimate group that has achieved success. Should it consider encouraging a larger membership and attendance at meetings or should it retain its current structure and identity?

- The IRSSD has always been a vehicle to carry the initial, small flicker of the flame of an idea where people have been able to express openly some very basic ideas. In the past, some of these flames have grown to become forest fires while others have simply died for whatever reason. Should the IRSSD continue to encourage the presentation of these initial flames or should it suggest that they wait until the flames are a little higher before being presented?

- Considering the contributions that have been made to this article and assuming (hypothetically!) that the IRSSD had $20 M (U.S.) to distribute among the various research areas, which areas would you support for receiving a contribution when thinking about the potential for most significant progress to be made?

- Considering the contributions that have been made to this article and assuming (hypothetically!) that the IRSSD had $20 M (U.S.) to distribute among the various research areas, which areas would you **NOT **support for receiving a contribution when thinking about the potential for most significant progress to be made?

- It might be argued that the driving force behind any research is application of the knowledge gained. In relation to the IRSSD, the application of the knowledge gained is aimed at helping the patient with spinal deformity. Recognising that practitioners (surgeons, physical therapists, chiropractors etc.) all help individual patients, what research completed under the umbrella of the IRSSD has affected the outcome of a single patient when it has been applied? (It is of interest to mention that the article entitled 'A patient's perspective' submitted by Joe O'Brien was added after all other aspects of the paper were finished, including this summary. The article speaks for itself about the IRSSD.)

Hopefully the contributions made in the various areas by the authors in this paper and the questions asked at the end provide stimulus for more exciting discussion at the upcoming meeting in Montreal in July 2010. We hope to see you there!

## Competing interests

The authors declare that they have no competing interests.

## Authors' contributions

Each author contributed equally to the writing of the article. Namely KMB contributed with the Introduction, VJR with the History of the IRSSD, MA with Motivating Clinical Problems, TBG with Surgical methods, IAS with General Biomechanics RGB and PHD with Aetiopathogenesis of adolescent idiopathic scoliosis (AIS), DH Evolution of measurement techniques, EL with Bracing for Scoliosis, HRW with Non-operative aspects and conservative treatment (including exercise and postural control techniques) of spinal disorders. TE, NA, AM with Metabolic and Hormonal Determinants of Adolescent Idiopathic Scoliosis, CEA with Finite Element Modeling of Scoliosis, JPO'B with The Value of the IRSSD ~A Patient's Perspective, KMB with Summary and Future.

All authors read and approved the final manuscript.

## Supplementary Material

Additional file 1 **Table 1**. International Research Society of Spinal Deformities (IRSSD) Scientific meetings and number of citations for the group of presented papers dedicated to the surgical treatment. Click here for file

Additional file 2 **Table 2**. Book references. Click here for file

Additional file 3 **"Non-operative aspects and conservative treatment"**. References for "Non-operative aspects and conservative treatment". Click here for file

Additional file 4 **"Metabolic and Hormonal Determinants of Adolescent Idiopathic Scoliosis"**. References for "Metabolic and Hormonal Determinants of Adolescent Idiopathic Scoliosis". Click here for file

