# Supplementary material for: The International Research Society of Spinal Deformities (IRSSD) and its contribution to science
Source: Scoliosis. 2009 Dec 22;4:28. doi: 10.1186/1748-7161-4-28 (PMC2808165; doi:10.1186/1748-7161-4-28)
Supplement: Additional file 1 — Table 1. International Research Society of Spinal Deformities (IRSSD) Scientific meetings and number of citations for the group of presented papers dedicated to the surgical treatment. [file 1748-7161-4-28-S1.DOC]

**Table 1**

**International Research Society of Spinal Deformities (IRSSD) Scientific meetings and number of citations for the group of presented papers dedicated to the surgical treatment**

Stockholm, Sweden June 1996 15 articles 07 citations*

Burlington, Vermont, USA, June 1998 04 articles 02 citations

Clermont Ferrand, France, May 2000 07 articles 09 citations

Athens, Greece, May 2002 25 articles 27 citations Vancouver, BC Canada, June 2004 17 articles 00 citations**

Ghent, Belgium, June 2006 19 articles 07 citations

Liverpool,UK July 2008 04 articles 00 citations***

* Google Scholar was the search tool for counting the citations of the articles published in the IOS Press Research into Spinal Deformities (RISD) in the “Studies in Health Technology and Informatics” (SHTI) book series.

** Unfortunately this book was not an IOS Press publication. Subsequently was not indexed in the Medline, therefore created 0 citations. The effect was that the research presented in this meeting was nearly lost due to the difficulty of availability of the book to the readers.

*** Too early for citations.
