# Supplementary material for: The International Research Society of Spinal Deformities (IRSSD) and its contribution to science
Source: Scoliosis. 2009 Dec 22;4:28. doi: 10.1186/1748-7161-4-28 (PMC2808165; doi:10.1186/1748-7161-4-28)
Supplement: Additional file 2 — Table 2. Book references. [file 1748-7161-4-28-S2.DOC]

**Table 2**

**Book references**

Research into Spinal Deformities 1 Volume 37 Studies in Health Technology and Informatics Edited by: J.S. Sevastik and K.M. Diab 1997, 524 pp hardcover ISBN: 90 5199 308 0 Price: US$122/€107/£75

Research into Spinal Deformities 2 Volume 59 Studies in Health Technology and Informatics Edited by: I.A.F. Stokes and P.H. Dangerfield 1999 523 pp Hardcover: ISBN: 90 5199 430 3 Price: US$107/€100/£64

Research into Spinal Deformities 3 Volume 88 Studies in Health Technology and Informatics Edited by: A. Tanguy and B. Peuchot 2002, 440 pp., hardcover ISBN: 1 58603 266 6 Price: US$115/€115/£73

Research into Spinal Deformities 4 Volume 91 Studies in Health Technology and Informatics Edited by: Th.B. Grivas 2002, 516 pp., hardcover ISBN: 1 58603 289 5 Price: US$115/€115/£72

International Research Society of Spinal Deformities, Symposium 2004, Editor Bonita J. Sawatzky, PhD, ISBN 0-88865-262-3. **

Research into Spinal Deformities 5 Volume 123 [Studies in Health Technology and Informatics](http://www.iospress.nl/html/shti.php) Edited by: D. Uyttendaele and P.H. Dangerfield June 2006, 640 pp., hardcover ISBN: 978-1-58603-630-0 Price: US$239 / €165 / £102

[Research into Spinal Deformities 6 (Edited by: P. Dangerfield)](http://www.iospress.nl/html/9781586038885.php) Research into Spinal Deformities 6 Volume 140 Studies in Health Technology and Informatics Edited by: P. Dangerfield September 2008, 400 pp., hardcover ISBN: 978-1-58603-888-5 Price: US$203 / €140 / £105
