# Supplementary material for: The International Research Society of Spinal Deformities (IRSSD) and its contribution to science
Source: Scoliosis. 2009 Dec 22;4:28. doi: 10.1186/1748-7161-4-28 (PMC2808165; doi:10.1186/1748-7161-4-28)
Supplement: Additional file 3 — "Non-operative aspects and conservative treatment". References for "Non-operative aspects and conservative treatment". [file 1748-7161-4-28-S3.DOC]

## References for “Non-operative aspects and conservative treatment”

Weiss HR, Negrini S, Hawes MC, Rigo M, Kotwicki T, Grivas TB, Maruyama T: **Physical exercises in the treatment of idiopathic scoliosis at risk of brace treatment – SOSORT consensus paper 2005.** *Scoliosis* 2006, **1:**6

Negrini S, Fusco C, Minozzi S, Atanasio S, Zaina F, Romano M: **Exercises reduce the progression rate of adolescent idiopathic scoliosis: Results of a comprehensive systematic review of the literature.**

*Disability & Rehabilitation* 2008, **30**(10)**:**772-785.

Weiss HR, Negrini S, Rigo M, Kotwicki T, Hawes MC, Grivas TB, Maruyama T, Landauer F: **Indications for conservative management of scoliosis (guidelines).**

*Scoliosis* 2006, **1**(1)**:**5.

Hawes M: **Impact of spine surgery on signs and symptoms of spinal deformity.**

*Pediatr Rehabil* 2006, **9**(4)**:**318-39

Wong MS, Cheng CY, Ng BK, Lam TP, Chiu SW**:** [**A comparison of the clinical effectiveness of spinal orthoses manufactured using the conventional manual method and CAD/CAM method in the management of AIS.**](http://www.ncbi.nlm.nih.gov/pubmed/17108431?itool=EntrezSystem2.PEntrez.Pubmed.Pubmed_ResultsPanel.Pubmed_RVDocSum&ordinalpos=5) Stud Health Technol Inform. 2006;123:225-32
