# Supplementary material for: The International Research Society of Spinal Deformities (IRSSD) and its contribution to science
Source: Scoliosis. 2009 Dec 22;4:28. doi: 10.1186/1748-7161-4-28 (PMC2808165; doi:10.1186/1748-7161-4-28)
Supplement: Additional file 4 — "Metabolic and Hormonal Determinants of Adolescent Idiopathic Scoliosis". References for "Metabolic and Hormonal Determinants of Adolescent Idiopathic Scoliosis". [file 1748-7161-4-28-S4.DOC]

**References for “Metabolic and Hormonal Determinants of Adolescent Idiopathic Scoliosis”**

Burwell, R. G., R. K. Aujla, et al. (2009). "Pathogenesis of adolescent idiopathic scoliosis in girls - a double neuro-osseous theory involving disharmony between two nervous systems, somatic and autonomic expressed in the spine and trunk: possible dependency on sympathetic nervous system and hormones with implications for medical therapy." Scoliosis **4**(1): 24.

Burwell, R. G., R. K. Aujla, et al. (2008). "Body mass index of girls in health influences menarche and skeletal maturation: a leptin-sympathetic nervous system focus on the trunk with hypothalamic asymmetric dysfunction in the pathogenesis of adolescent idiopathic scoliosis?" Stud Health Technol Inform **140**: 9-21.

Drummond, D. S. and E. J. Rogala (1980). "Growth and maturation of adolescents with idiopathic scoliosis." Spine (Phila Pa 1976) **5**(6): 507-11.

Dubousset, J. and M. Machida (2001). "[Possible role of the pineal gland in the pathogenesis of idiopathic scoliosis. Experimental and clinical studies]." Bull Acad Natl Med **185**(3): 593-602; discussion 602-4.

Inoue, M., S. Minami, et al. (2002). "Association between estrogen receptor gene polymorphisms and curve severity of idiopathic scoliosis." Spine (Phila Pa 1976) **27**(21): 2357-62.

Letellier, K., B. Azeddine, et al. (2008). "Estrogen cross-talk with the melatonin signaling pathway in human osteoblasts derived from adolescent idiopathic scoliosis patients." J Pineal Res **45**(4): 383-93.

Lonstein, J. E. and J. M. Carlson (1984). "The prediction of curve progression in untreated idiopathic scoliosis during growth." J Bone Joint Surg Am **66**(7): 1061-71.

Lowe, T., D. Lawellin, et al. (2002). "Platelet calmodulin levels in adolescent idiopathic scoliosis: do the levels correlate with curve progression and severity?" Spine (Phila Pa 1976) **27**(7): 768-75.

Lowe, T. G., R. G. Burwell, et al. (2004). "Platelet calmodulin levels in adolescent idiopathic scoliosis (AIS): can they predict curve progression and severity? Summary of an electronic focus group debate of the IBSE." Eur Spine J **13**(3): 257-65.

Machida, M., J. Dubousset, et al. (1996). "Melatonin. A possible role in pathogenesis of adolescent idiopathic scoliosis." Spine (Phila Pa 1976) **21**(10): 1147-52.

Machida, M., J. Dubousset, et al. (2006). "Experimental scoliosis in melatonin-deficient C57BL/6J mice without pinealectomy." J Pineal Res **41**(1): 1-7.

Machida, M., I. Murai, et al. (1999). "Pathogenesis of idiopathic scoliosis. Experimental study in rats." Spine (Phila Pa 1976) **24**(19): 1985-9.

Moreau, A., D. S. Wang, et al. (2004). "Melatonin signaling dysfunction in adolescent idiopathic scoliosis." Spine (Phila Pa 1976) **29**(16): 1772-81.

Qiu, Y., X. Sun, et al. (2007). "Decreased circulating leptin level and its association with body and bone mass in girls with adolescent idiopathic scoliosis." Spine (Phila Pa 1976) **32**(24): 2703-10.

Quinton, N. D., R. F. Smith, et al. (1999). "Leptin binding activity changes with age: the link between leptin and puberty." J Clin Endocrinol Metab **84**(7): 2336-41.

Thomas, T., B. Burguera, et al. (2001). "Role of serum leptin, insulin, and estrogen levels as potential mediators of the relationship between fat mass and bone mineral density in men versus women." Bone **29**(2): 114-20.

Zhong, N., X. P. Wu, et al. (2005). "Relationship of serum leptin with age, body weight, body mass index, and bone mineral density in healthy mainland Chinese women." Clin Chim Acta **351**(1-2): 161-8.
